# A Comparison of Basic Training Variables in the Standard and Cambered Bar Bench Press Performed to Volitional Exhaustion

**DOI:** 10.5114/jhk/162516

**Published:** 2023-04-20

**Authors:** Patryk Matykiewicz, Michał Krzysztofik, Adam Zając

**Affiliations:** 1 Institute of Sport Sciences, The Jerzy Kukuczka Academy of Physical Education in Katowice, Katowice, Poland.

**Keywords:** ROM, fatigue, movement velocity

## Abstract

The objective of this study was to compare the impact of cambered and standard barbells used during the bench press exercise on the number of performed repetitions and mean velocity during a bench press training session that included 5 sets performed to volitional failure at 70% of one-repetition maximum (1RM) (for each barbell type). An additional objective was to determine whether there would be any difference in neuromuscular fatigue assessed by peak velocity changes during bench press throws performed 1 and 24 hours after the cessation of each session. The research participants included 12 healthy resistance-trained men. Participants performed 5 sets of the bench press exercise to volitional failure against 70% of 1RM with the cambered or standard barbell. The Friedman’s test showed an overall trend of a significant decrease in the mean velocity (p < 0.001) and a number of performed repetitions (p < 0.001) from the first to the fifth set (p < 0.006 and p < 0.02, respectively for all) under both conditions, yet neither bar showed significant differences between the corresponding sets. Two-way ANOVA indicated a significant main effect of time (p < 0.001) for peak velocity during the bench press throw. The post-hoc comparisons showed significantly lower peak velocity during the bench press throw one hour after the bench press compared to pre (p = 0.003) and 24-hour post intervention (p = 0.007). Both barbells caused a similar decrease in peak barbell velocity during the bench press throw performed one hour after the bench press training session, with values returning to baseline 24 hours later. This indicates that bench press workouts with either a standard or a cambered barbell present the same training demands.

## Introduction

The bench press exercise is perhaps one of the most popular resistance exercises for developing upper-body strength, power and hypertrophy (Krol and Golas, 2017; Shoenfeld et al., 2015). It is also often used for research and testing (Stastny et al., 2017). The correct technique of the bench press requires the athlete to lower the barbell to the chest and then press upwards until the elbows are fully extended (Gomo and Van Den Tillaar, 2016). However, the barbell bench press is one of the few exercises in which the entire physiological range of motion (ROM) of the prime movers (in this case, the pectoralis major, anterior deltoid, and triceps brachii) is not fully achieved because the athlete is limited by the barbell (Lockie et al., 2017). Specifically, during the standard barbell bench press, the “full” ROM is limited by the shape of the barbell, which touches the chest. Equipment known as the cambered barbell has been created to eliminate this restriction. The cambered barbell's U-shape provides greater torso room and allows to reach a lower-end position of the barbell in the bench press movement in comparison to the standard barbell (Matykiewicz et al., 2021). One of the cambered bar's tenets is to help athletes extend their chest and shoulder muscles to a greater extent during the bottom phase of the bench press movement (Corey, 1991).

Previous studies have already compared the impact of cambered and standard barbell bench presses on muscle activity (Krzysztofik et al., 2020a), barbell velocity (Krzysztofik et al., 2020b; Matykiewicz et al., 2021), post-activation performance enhancement (Krzysztofik et al., 2022), and training volume (Krzysztofik et al., 2021). Krzysztofik et al. (2020a) have revealed that the cambered barbell leads to greater activation of the anterior deltoid, while the standard barbell causes higher pectoralis major and triceps brachii long head activity during the bench press exercise at 90% of the one-repetition maximum (1RM). Additionally, the cambered barbell significantly enhances power output and bar velocity in the bench press exercise at 50% of 1RM compared with the standard barbell, according to studies by Krzysztofik et al. (2020b) and Matykiewicz et al. (2021). On the other hand, the standard bar bench press turned out to be superior as a conditioning activity to acutely enhance bench press throw performance compared with the cambered bar (Krzysztofik et al., 2022). Finally, Krzysztofik et al. (2021) evaluated the effects of 3 sets of bench presses with a standard or a cambered barbell until volitional failure at 50% of 1RM on training volume and peak barbell velocity. However, those studies were limited by testing protocols, which seem unusual when compared to regular strength training workouts, that is, performing a single repetition at 50, 70, and 90%1RM (Krzysztofik et al., 2020a), a single set of 3 repetitions at 50%1RM (Krzysztofik et al., 2020b) or 3 sets of 3 repetitions at 50%1RM (Matykiewicz et al., 2021). To the best of the authors' knowledge, only one study has examined a higher-volume cambered barbell bench press session (Krzysztofik et al., 2021). In addition, none of those studies were designed to compare the effects of bench presses on immediate and delayed fatigue, despite the fact that the ROM may have an impact on its magnitude. However, in the study by Krzysztofik et al. (2021), changes in barbell velocity, which is considered an indicator of neuromuscular fatigue (de-Oliveira et al., 2022; Sánchez-Medina and González-Badillo, 2011), were assessed. Those authors compared the velocities obtained during successive sets of standard and cambered barbell presses until failure and found a similar decrease in peak velocity from set to set. However, the magnitude of differences in velocity between cambered and standard barbells was large in the first set (effect size g = 1.14), while it was medium in the second and third one (g = 0.53–0.6). Moreover, a greater enhancement of bench press throw performance after a standard barbell than a cambered barbell bench press was reported by Krzysztofik et al. (2022), what may also indicate that higher neuromuscular fatigue was induced by a standard barbell. Therefore, this may suggest that a greater ROM and stretch achieved during a cambered barbell bench press may induce a higher level of fatigue, followed by a prolonged recovery process. These results indicate that cambered bar bench press training may require significant adjustments of particular training variables, such as the rest intervals, the number of sets and training frequency.

Considering that the interaction of particular training variables, such as training volume, intensity, rest intervals, muscle action, and ROM, greatly influence the magnitude of fatigue imposed by strength training (Toigo and Boutellier, 2006), the aim of this study was to compare differences in cambered and standard barbell bench presses in training volume and mean barbell velocity across 5 sets of this exercise performed until volitional failure at 70%1RM and its impact on neuromuscular fatigue assessed by changes in peak velocity during the bench press throw performed 1 h and 24 h later. We hypothesized that the use of the cambered barbell would allow for greater velocities during the bench press with no significant changes in training volume, but would induce a higher level of fatigue in comparison to the standard barbell due to the significantly greater ROM.

## Methods

### Participants

Twelve male resistance-trained adults were recruited for this study (Table 1). The inclusion criteria were as follows: no musculoskeletal injuries prior to the investigation, a minimum of 5 years of resistance training experience, 1RM bench press of at least 100% of own body mass. Additionally, to avoid the influence of the learning effect on the research outcomes, four weeks of prior experience with the cambered bar bench press exercise were also required. All participants signed an informed consent form after receiving information about the study's objectives, methods, potential advantages, and risks. All measurements were conducted in the Strength and Power Laboratory of the Academy of Physical Education in Katowice, Poland. All procedures followed the most recent edition of the Declaration of Helsinki, 2013, and the research protocol was approved by the Bioethics Committee for Scientific Research of the Academy of Physical Education in Katowice, Poland (3/2021).

**Table 1:** Descriptive characteristics of participants.

Age [years]	25.9 ± 4.2
Body Mass [kg]	88 ± 9.1
Height [cm]	178.3 ± 5.3
Experience [years]	9.8 ± 4.7
Standard bar 1RM [kg]	132 ± 21
Cambered bar 1RM [kg]	126 ± 20
Standard bar ROM [cm]	35 ± 3.7
Cambered bar ROM [cm]	38.9 ± 2.9

1RM – one repetition maximum; ROM – range of motion

### Measures

**Figure 1:**
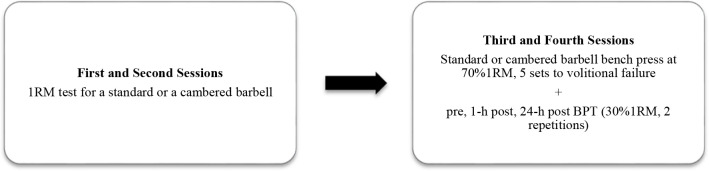
Schematic representation of the experimental protocol. 1RM – one-repetition maximum

All participants performed both, the cambered and standard bench press training sessions, which were carried out using a randomized crossover design. This setup aimed to investigate the effects of particular bench press sessions on subsequent and delayed bench press throw performance. Two familiarization and two experimental sessions were attended by all participants. One of these sessions included a one-repetition maximum test of the flat bench press (1RM test) with a standard and a cambered barbell. The experimental procedures consisted of 5 sets of the bench press exercise with a load equal to 70% of the standard or the cambered bar bench press 1RM to volitional failure (Figure 1). The 1RM tests were performed 72 h apart, while the bench press sessions were executed one week apart. To prevent fatigue, participants were instructed to avoid additional resistance exercise within 72 hours of testing.

### Design and Procedures

The next two sessions were identical except for the use of a standard or a cambered barbell during the bench press session. During particular sessions, participants performed 5 sets of the bench press exercise to momentary volitional failure with either a standard or a cambered barbell at a load equal to 70% of the participants’ 1RM (specific bar), in randomized order. The bench press tempo of movement was identical to the 1RM test and a 5-min rest interval was introduced between each set. To prevent circadian fluctuation, all tests were carried out at the same time of the day (12:00 and 15:00 pm), and were separated by a 96 h recovery period. Since the movement velocity has been previously shown as an indicator of neuromuscular fatigue (Sánchez-Medina and González-Badillo, 2011), changes in barbell velocity during the bench press throw (BPT) were evaluated to determine how each barbell bench press session affected the time course of fatigue. For this purpose, prior to and 1 h, as well as 24 h after each session, all participants performed a single set of two repetitions of the BPT on the Smith machine at maximal velocity against a load of 30% of 1RM of the standard barbell BP. Moreover, mean velocity, as well as barbell displacement, and the number of performed repetitions, were recorded during each set of the bench press exercise. Peak velocity was measured during the BPT. A Tendo Power Analyzer system (Tendo Sport Machines, Trencin, Slovakia) was used for measuring bar velocity and displacement during both the bench press exercise and the bench press throw (Drinkwater et al., 2007).

### One-Repetition Maximum Bench Press Test

The first two sessions aimed to determine the 1RM either with a standard or a cambered barbell. Each experimental session began with the standard warm-up detailed elsewhere (Matykiewicz et al., 2021). Afterwards, using a standard or a cambered bar, participants performed the 1RM bench press test. They performed a single repetition, without pausing, with a constant tempo of the eccentric phase of the movement (2 s) and a volitional tempo of the concentric phase of the lift (Wilk et al., 2020). Hand positioning on the bar was similar throughout each trial and was placed at 150% of the participant's bi-acromial distance (Green and Comfort, 2007). The test consisted of three to five attempts. The first attempt was set at 80% of the self-reported 1RM, and if successfully lifted, the weight was increased by 2.5 kg to 5 kg in the following attempts. Participants were instructed about BP technique requirements, which included keeping the feet on the floor, hips in contact with the bench, and not bouncing the barbell off the chest. Two experienced spotters were present at all times to guarantee safety.

### Statistical Analysis

SPSS software (version 25.0; SPSS, Inc., Chicago, IL, USA) was used to perform all statistical analyses, and data are presented as means with standard deviations (± SD). Statistical significance was set at *p* < 0.05. The normality of data distribution was checked using the Shapiro-Wilk test, while the Mauchly's test was used to check the assumption of sphericity. Two-way ANOVA or, if the normality was not confirmed, the related-samples Friedman’s two-way ANOVA by ranks was used to investigate differences in training variables during standard and cambered barbell bench presses and their influence on the bench press throw performance. Pairwise comparisons were examined using post-hoc tests with Bonferroni correction when a significant main effect or interaction was found. Standardized effect sizes were used to express the size of mean differences. Thresholds for qualitative descriptors of Hedges g were interpreted as small, medium, and large at ≤20, 0.21–0.79 and >0.80, respectively (Cohen, 2013).

## Results

The Shapiro-Wilk tests indicated a violation of data distribution for the following variables: the number of performed repetitions, mean velocity during the bench press, and peak power during the bench press throw.

The t-test showed a significantly higher 1RM value in the standard than in the cambered bench press (132 ± 21 kg vs. 126 ± 20 kg; *p* < 0.001; ES = 0.40).

### Number of Performed Repetitions during the Bench Press

The t-test showed a significantly higher total number of performed repetitions during the standard than the cambered bench press exercise (49 ± 7 vs. 43 ± 8; *p* = 0.005, ES = 0.77).

**Figure 2:**
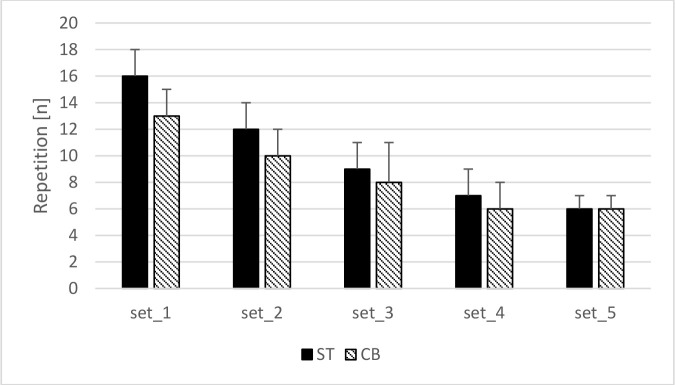
Differences in the number of performed repetitions during the standard and cambered barbell bench press. ST – standard barbell bench press, CB – cambered barbell bench press

The Friedman’s test (test = 94.913; *p* < 0.001; Kendall’s W = 0.879) showed an overall trend of a significant decrease in the number of performed repetitions from the first to the fifth set (*p* < 0.02 for all) under both conditions, yet no significant differences between corresponding sets of standard and cambered bench presses were observed (Figure 2).

### Range of Motion during the Bench Press

The t-test showed no significant difference in total load displacement between the cambered and the standard barbell bench press (1698 ± 286 cm vs. 1771 ± 382 cm; *p* = 0.308, ES = 0.21).

**Figure 3:**
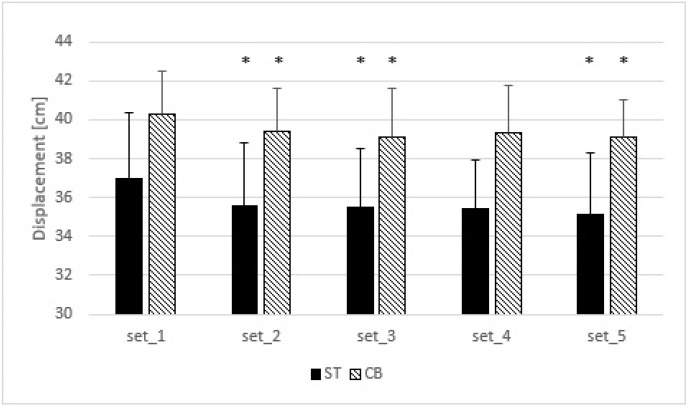
Differences in the ROM during the standard and cambered barbell bench press. * significant difference in comparison to the first set ST – standard barbell bench press, CB – cambered barbell bench press

Two-way ANOVA indicated a non-significant interaction (F = 0.583; *p* = 0.567; η^2^ = 0.05), but a significant main effect of condition (F = 11.286; *p* = 0.006; η^2^ = 0.506) and set (F = 12.243; *p* = 0.001; η^2^ = 0.527). The post-hoc comparisons showed significantly greater ROM during the cambered than the standard barbell bench press (*p* = 0.006; ES = 1.34). Moreover, the ROM was significantly greater in the first set compared to the second (*p* = 0.33; ES = 0.35), third (*p* = 0.28; ES = 0.41), and fifth sets (*p* = 0.004; ES = 0.47) (Figure 3).

### Mean Velocity during the Bench Press

**Figure 4:**
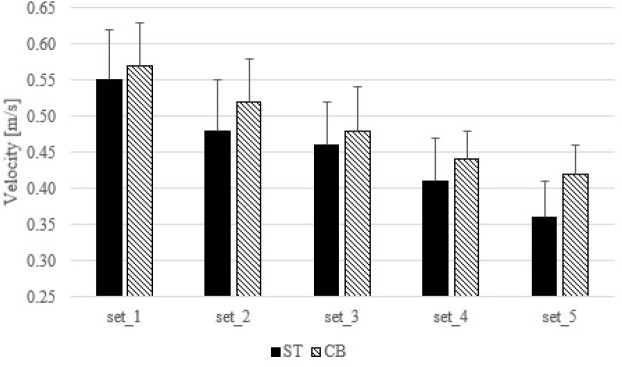
Comparison of mean velocity during the standard and cambered barbell bench press. ST – standard barbell bench press, CB – cambered barbell bench press

The Friedman’s test (test = 74.205; *p* < 0.001; Kendall’s W = 0.687) showed an overall trend of a significant decrease in mean velocity from the first to the fifth set (*p* < 0.006 for all) under both conditions, however, no significant differences between corresponding sets of the standard and cambered bench presses were noted (Figure 4).

### Peak Velocity during the Bench Press Throw

**Figure 5:**
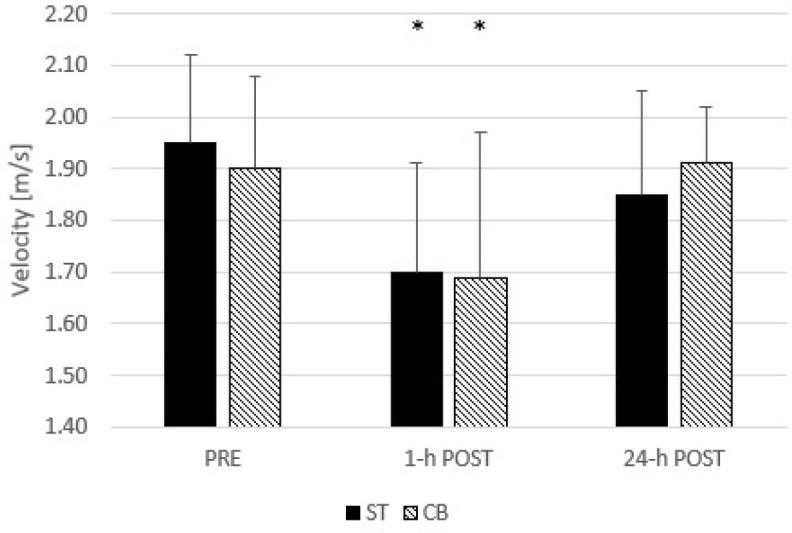
Changes in peak velocity during the bench press throw at pre, 1-h post, and 24-h post the standard and the cambered barbell bench press session. * significant difference in comparison to the pre ST – standard barbell bench press, CB – cambered barbell bench press

Two-way ANOVA indicated a non-significant interaction (F = 1.374; *p* = 0.274; η^2^ = 0.111) and a main effect of condition (F = 0.012; *p* = 0.914; η^2^ = 0.001), but a significant main effect of time points (F = 14.721; *p* < 0.001; η^2^ = 0.572). Post-hoc comparisons showed significantly lower peak velocity 1-h post bench press compared to pre (*p* = 0.003; ES = 1.11) and 24-h post intervention (*p* = 0.007; ES = 0.9) (Figure 5).

## Discussion

The main aim of this study was to compare the impact of cambered and standard barbells used during the bench press exercise on the number of performed repetitions and mean velocity during a bench press training session that included 5 sets performed to volitional failure at 70% of 1RM (for each barbell). An additional objective was to determine whether there would be any difference in neuromuscular fatigue assessed by peak velocity changes during bench press throws performed 1 and 24 hours after the end of each session. The results showed a significantly greater ROM during the cambered than the standard barbell bench press, as well as a gradual decrease in the number of performed repetitions and barbell velocity across the sets, but no significant differences between the barbells in these variables. Moreover, under both conditions, a similar significant decrease in barbell velocity during the bench press throw 1 hour after the training session was found, but 24 hours later, no significant changes in comparison to baseline values were reported. Furthermore, participants lifted a significantly higher maximal load in the standard bar when compared with the cambered one, confirming that the greater ROM of exercise results in a lower 1RM (Martínez-Cava et al., 2019, 2022; Pallarés et al., 2020).

To date, research comparing the impact of cambered and standard barbells during bench press training has focused on differences in electromyographic activity, power output, and barbell velocity (Krzysztofik et al., 2020a, 2020b; Matykiewicz et al., 2021). These studies have consistently shown that a cambered barbell contributes to a significantly higher ROM during the bench press exercise, which leads to significantly higher barbell velocities (Krzysztofik et al., 2021; Matykiewicz et al., 2021). However, to the best of the authors' knowledge, the current study is the second one to date (Krzysztofik et al., 2021) that compared the impact of a cambered barbell on changes in the number of performed repetitions and barbell velocity in the protocol, imitating a bench press training workout to a greater extent than procedures of previous studies. Although in the current study, more sets (5 vs. 3) were performed and a higher load (70% 1RM vs. 50% 1RM) was used compared to the previous study (Krzysztofik et al., 2021), the results were similar and showed a significantly lower total number of repetitions in the bench press workout performed with the cambered barbell compared to the standard one, with no differences in load displacement. Considering the above, evaluating only the number of performed repetitions may lead to an incorrect estimation of training volume due to the exclusion of variations in the ROM (Krzysztofik et al., 2021). As a result, despite the fact that the cambered barbell allows for a significant increase in ROM during the bench press exercise, it has no effect on the volume of the bench press training workout as measured by load displacement. On the other hand, more repetitions indicate that the eccentric-concentric transition phase was performed more often, which results in higher peak torque output and greater mechanical stress that may differ in magnitude to the elicited fatigue and training stimuli (Chapman et al., 2006). These may be the major training implications considering that exercise volume, calculated as the number of performed repetitions, is a key factor in chronic adaptations related to muscle hypertrophy (Kraemer and Ratamess, 2004; Schoenfeld et al., 2019). Nonetheless, the available literature provides premises that a greater ROM confers beneficial effects on muscle hypertrophy and strength adaptations in comparison to partial ones (Martínez-Cava et al., 2022; Schoenfeld and Grgic, 2020). For example, Martinez-Cava et al. (2022) showed greater gains in muscle strength after 10 weeks of standard barbell bench press training performed at full ROM compared to partial ROM. Therefore, it is possible to conclude that while performing bench press training sessions with a cambered or a standard bar, various adaptive changes may occur. It seems that further research is needed that would expand the knowledge of long-term use of the cambered bar in bench press training.

The fact that there were no significant differences in mean bar velocity during the bench press exercise between the two types of bars is another finding from the current study that needs to be emphasized. These findings contradict earlier research that found significantly lower bench press bar velocities in a smaller ROM (Martínez-Cava et al., 2019). Also, studies comparing cambered and standard barbell velocities during the bench press (Krzysztofik et al., 2020b, 2021; Matykiewicz et al., 2021) showed higher velocity values during a cambered barbell bench press. It should be noted, however, that the procedures of those studies differed significantly. In the study by Krzysztofik et al. (2021), the average of peak velocities was obtained during sets performed until volitional failure at 50% of 1RM. As a consequence, significantly higher mean bar velocity was observed while using a cambered compared to a standard bar in a study by Krzysztofik et al. (2020b), however, participants only performed 3 repetitions at 50% 1RM. On the other hand, in the current study, the average of mean velocities in a given set performed to volitional failure at 70% of 1RM was measured. Nonetheless, this study found a slightly higher barbell velocity during the cambered bench press compared to the standard barbell bench press, though this difference did not reach statistical significance. It should also be emphasized that the peak velocity measure in the study by Krzysztofik et al. (2021) is the instantaneous value of velocity, the fastest single moment during the entire concentric phase of the movement. The mean velocity, which represents the average velocity for the entire concentric phase of the movement, may provide more comprehensive information to assess training demands due to traditional resistance exercises performed until failure. The current study's findings revealed a similar trend of decreasing velocity from set to set with no difference between barbells. Considering that previous research on resistance training has shown that velocity loss may objectively quantify neuromuscular fatigue (de-Oliveira et al., 2022; Sánchez-Medina and González-Badillo, 2011), it indicates that bench pressing with either a cambered or a standard barbell contributes to a similar increase in fatigue with each successive set. This confirms that using a cambered barbell may not require longer rest intervals between sets.

Considering the fact that training frequency is another variable affecting adaptations to resistance training (Kraemer and Ratamess, 2004), this study also aimed to determine whether training with a cambered barbell would contribute to greater fatigue, which may negatively affect successive training sessions. As it turned out, both the standard and the cambered bar bench press exercise caused a considerable drop in peak velocity one hour after the training session, but 24 hours later, there were no significant differences in neuromuscular performance evaluated by the BPT. This is another aspect that indicates the lack of significant differences between bench press training with a standard and a cambered bar. This indicates, in conjunction with the lack of differences in load displacement and velocity, that training with a cambered barbell may not require a different training volume, intensity, or frequency approach. However, it should be mentioned that 24 h after the training session with the standard barbell, velocity was still slightly reduced, but this value did not reach the level of statistical significance (g = 0.36). Therefore, to comprehensively assess the consequences of cambered barbell bench press training, further studies should examine changes in fitness, and also in muscle damage markers.

Some limitations should be considered when drawing conclusions from this study. First, participants performed 5 sets to voluntary failure, which is not a typical training approach, thus further studies should compare bench press training sessions with a given number of sets and repetitions at a fixed load. Furthermore, only peak velocity during the bench press throw was used to assess neuromuscular fatigue, which is clearly insufficient to determine the true physiological disturbance; thus, additional studies should measure, i.e., muscle damage markers. Bench press throw performance was only measured up to 24 hours after the intervention, despite the fact that fatigue symptoms may last longer. Additionally, the subjective level of physical exertion was not evaluated.
